# Human Values Across the Lifespan: Age-Graded Differences at Three Hierarchical Levels and What We Can Learn From Them

**DOI:** 10.1177/01461672241312570

**Published:** 2025-02-05

**Authors:** Andrés Gvirtz, Matteo Montecchi, Amy Selby, Friedrich M. Götz

**Affiliations:** 1 King’s College London, London, UK; 2 University of Cambridge, Cambridge, UK; 3 The University of British Columbia, Vancouver, Canada

**Keywords:** Schwartz values, value hierarchy, value nuances, integrative modeling

## Abstract

Personality-development research is flourishing. Here, we extend these efforts horizontally (new constructs) and vertically (new levels within the same construct) by charting out age-graded differences in Schwarz’s human values across 80,814 individuals. Conducting a systematic investigation of cross-sectional age-graded differences in human values—from late teenage years to post-retirement—featuring 36 analytical model choices and 180,000 simulation-based decisions, our analyses replicate some earlier findings (e.g., increasing self- and growth-focus during adolescence and increasing security concerns during adulthood), while also highlighting complex and previously unappreciated dynamics. As such, while it is a common practice to aggregate specific values into parsimonious higher-order concepts to ease interpretation, this may risk overlooking meaningful trends in lower-order value development. Specifically, revealing unique and asynchronous patterns for value nuances, we find that aggregation (a) leads to a loss of critical information, (b) creates conflicting results when nuances diverge, and (c) significantly reduces predictive power.

## Introduction

Over the past two decades, personality development has evolved into a frontrunner of the field and one—if not the—major driving force leading the renaissance of personality science (Roberts & Yoon, 2022). Co-occurring with the increasing availability of large-scale data that allow researchers to trace human personality development (Roberts & Yoon, 2022), a fast-growing base of evidence is providing ample support for both stability and continuous plasticity of personality across the lifespan. This base of evidence is powered both by longitudinal datasets that assess within-person change and large-scale cross-sectional datasets that assess age-graded between-person differences (as in the present work), which have tended to produce largely consistent evidence (Roberts & Yoon, 2022).^1^ Specifically, while recognizing cultural (Bleidorn et al., 2013) and individual (Graham et al., 2020; Schwaba et al., 2023) differences in the rate, timing, and direction of personality change, as well as some variation in observed developmental trends across different personality measures of the same traits (Costa et al., 2019), this body of work suggests several common developmental patterns. That is, during adolescence, humans tend to undergo a period of antagonism and rebellion—often referred to as the disruption hypothesis (Denissen et al., 2013; Soto & Tackett, 2015). As people enter adulthood, they typically become more well-adjusted, responsible, communal, and emotionally stable (Bleidorn, 2015; Bleidorn et al., 2013; Roberts et al., 2008)—a pattern known as the maturity principle (Bleidorn et al., 2013; Roberts et al., 2008). Continuous albeit less-drastic changes appear to occur throughout middle age (Kandler et al., 2015). While less is known about old age, there is some evidence that individuals tend to regress at that life stage (Graham et al., 2020; Mõttus et al., 2012; Wagner et al., 2016)—a pattern that has been described as a reversal of personality maturation (Bleidorn & Hopwood, 2019). Reflecting their pre-eminent position in the field of personality psychology as a whole, most of the work in personality development has focused on the Big Five trait domains (Bleidorn et al., 2021; Costa et al., 2019; Specht, 2017)—that is, Openness to Experience, Conscientiousness, Extraversion, Agreeableness, and Neuroticism (OCEAN for short; Costa & McCrae, 1992; Digman, 1990; John, 2021). However, just like there is more to personality than the Big Five domains, there is also more to personality development.

### Horizontal and Vertical Expansions of the Personality-Development Literature

Consistent with a push to move beyond the “Big Few” (Mõttus et al., 2020), new work is actively expanding the scope of the personality-development literature both horizontally and vertically. In keeping with a broad understanding of personality as subsuming any form of relatively stable psychological differences between people, regardless of their content and breadth (Baumert et al., 2017; Rauthmann, 2020), horizontal extensions have begun to describe lifespan development of other personality constructs such as narrative identity (McAdams & Olson, 2010), subjective well-being (Luhmann, 2017), or Machiavellianism (Götz, Bleidorn, & Rentfrow, 2020). Meanwhile, vertical extensions have stayed within the Big Five taxonomy but started to explore age-related trends in the more narrowly-defined lower-level elements of the Big Five. Zooming in on Big Five facets, a number of studies have demonstrated that more differentiated patterns emerge as trait specificity increases, hence offering a more complete understanding of personality development (Jackson et al., 2009; Roberts et al., 2006; Schwaba et al., 2022; Soto et al., 2011; Soto & John, 2012). Encouraged by this, recent studies have argued—and shown—that even more age-related information is available at the lowest, most granular level of the Big Five hierarchy (Hang et al., 2021; Mõttus & Rozgonjuk, 2021). This level has been termed personality nuances (Condon et al., 2020; McCrae, 2015; McCrae & Mõttus, 2019; Mõttus et al., 2017), is often understood as comprising the most basic building blocks of the personality trait hierarchy (Achaa-Amankwaa et al., 2021; Seeboth & Mõttus, 2018), and is commonly operationalized through single personality items (Hang et al., 2021; Mõttus, 2016; Mõttus et al., 2017; Seeboth & Mõttus, 2018). The additional developmental information afforded at the nuance level is considerable. Nuances have been found to contain over 40% more age-related information than facets and over 130% more age-related information than the Big Five domains (Mõttus & Rozgonjuk, 2021). Importantly, revealing nuance-specific associations is not just a technical exercise but may be the key to (a) reconciling inconsistent findings across studies (due to different personality measurements and, in turn, different nuance sampling), and (b) achieving a better understanding of personality development that is at once more holistic and more specific (Hang et al., 2021). Thus, an intuitive next step for the field of personality development would be to combine horizontal and vertical extensions by examining age-graded changes in constructs beyond the Big Five at different hierarchical levels, with varying granularity. Indeed, initial evidence from intelligence-development research suggests that the notion of nuances as meaningful and information-rich conceptual units can also be fruitfully applied to other personality variables (Schroeders et al., 2021). Building on this, in the current research, we adopt a hierarchical approach to the investigation of personality development. That is, we examine age-graded differences in personal values across the lifespan at three hierarchically stacked levels (from least to most granular): higher-order values, basic values, and value nuances.

### Personal Values and Their Development Across the Lifespan

Values are broad, trans-situational goals and represent guiding principles in people’s lives that reflect what is important and desirable to them (Rokeach, 1973; Sagiv et al., 2017; Schwartz, 1992). Values are a core component of the self (Hitlin, 2003; Sagiv & Schwartz, 2022) and human personality (Rauthmann, 2020) and affect a wide range of consequential emotions, cognitions, perceptions, attitudes, behaviors, and life outcomes (Sagiv & Roccas, 2021), from religiosity (Roccas & Elster, 2014; Saroglou et al., 2004; Schnabel & Grötsch, 2015), pro-sociality (Arieli et al., 2020; Bardi & Schwartz, 2003; Maio et al., 2009), and self-esteem (Du et al., 2023; Fetvadjiev & He, 2019; Grosz et al., 2021), via aggression (Benish-Weisman et al., 2017), ethical transgressions (Feldman et al., 2015; Pulfrey & Butera, 2013), and delinquent behaviors (Aquilar et al., 2018; Bilsky & Hermann, 2016; Liu et al., 2007) to voting (Aspelund et al., 2013; Caprara et al., 2006, 2017), political activism (Roets et al., 2014; Sanderson & McQuilkin, 2017; Vecchione et al., 2015), and career choices (Arieli et al., 2016; Gandal et al., 2005; Knafo & Sagiv, 2004).

The predominant, most empirically validated value theory (Knafo et al., 2011; Maio, 2010; Rohan, 2000; Sagiv & Roccas, 2021) is Schwartz’s circumplex model (Schwartz, 1992, 1994, 2012). The model comprises four higher-order value constructs, which—in turn—are composed of 10 basic values. Reflecting the interrelated nature of values, the model also embeds two basic value conflicts (Sagiv & Roccas, 2021; Sagiv & Schwartz, 2022). The first conflict (personal versus social) contrasts the higher-order value *self-enhancement* (comprising the basic values *power* and *achievement*) with the higher-order value *self-transcendence* (comprising the basic values *benevolence* and *universalism*). The second conflict (self-expansion versus self-protection) contrasts the higher-order value *openness to change* (comprising the basic values *self-direction* and *stimulation*) with the higher-order value *conservation* (comprising the basic values *tradition, conformity*, and *security*). The tenth basic value, *hedonism*, shares elements of both *self-enhancement* and *openness to change*. This intermediate layer is measured by 20 individual items (Sandy et al., 2017), which—following previous Big Five research (Hang et al., 2021; Mõttus et al., 2017; Seeboth & Mõttus, 2018)—we parse into 20 granular value nuances, thus arriving at the most granular level of value conceptualization (see Table 1 for an overview). The cross-cultural utility of Schwartz’s model has been affirmed in over 500 samples across almost 100 countries (Bilsky et al., 2011; Davidov et al., 2008; Sagiv & Schwartz, 2022; Schwartz & Cieciuch, 2022).

**Table 1.** Overview of the Schwartz Human Values as Measured Through the Twenty-Item Values Inventory (TwIVI).

Higher-order values	Basic values	Value nuance
1. Openness to change	1. Self-direction	1. Curiosity
		2. Creativity
	2. Stimulation	3. Risk-taking
		4. New things
	3. Hedonism^a^	5. Have fun
		6. Enjoy life
2. Self-enhancement	4. Achievement	7. Getting ahead
		8. Being successful
	5. Power	9. Leader
		10. Be in charge
3. Conservation	6. Security	11. Organized
		12. Stable government
	7. Conformity	13. Respect
		14. Behave properly
	8. Tradition	15. Religion
		16. Tradition
4. Self-transcendence	9. Benevolence	17. Help people
		18. Needs of others
	10. Universalism	19. Treating people equal
		20. Harmony

*Note*. Adapted from the work of Sandy et al. (2017). Each question is assessed on a six-point Likert-type scale, ranging from “not like me at all” to “very much like me.”

a While hedonism is sometimes considered to be part of *openness to change* as well as *self-enhancement*, factor loadings show a much stronger alignment with *openness to change*, leading to us assigning it to *openness to change*, in line with previous research (Schwartz, 2012).

Despite the prominent position of personal values in personality psychology and their substantial and multi-faceted effects on how humans live (Sagiv et al., 2017; Sagiv & Roccas, 2021; Sagiv & Schwartz, 2022), relatively few studies have examined their development across the lifespan (Bardi et al., 2014; Borg, 2021). While existing studies do not always produce consistent results and may not allow for a particularly fine-grained and nuanced perspective, a few general age trends emerge. First, in line with the disruption hypothesis (Denissen et al., 2013; Soto & Tackett, 2015), during adolescence, individual value priorities become more self- and growth-focused and less other- and protection-focused (Daniel & Benish-Weisman, 2019; Sagiv & Schwartz, 2022; Vecchione et al., 2020) as reflected in increases in *self-enhancement* and *openness to change* values and decreases or stagnation in *self-transcendence* and *conservation* values. More recent studies emphasize the role of sociodemographic factors such as gender (Smallenbroek & Stanciu, 2024), generational cohort (Leijen et al., 2022), and level of education (Smallenbroek et al., 2023) in shaping trajectories of value development. For instance, while some values remain relatively stable across generational cohorts, others—such as hedonism—are particularly emphasized by Millennials compared to other groups (Leijen et al., 2022). Second, consistent with the maturity principle (Bleidorn et al., 2013; Roberts et al., 2008), this pattern reverses with adulthood, characterized by a growing emphasis on social (versus personal) and security (versus growth) concerns (Borg, 2021; Dobewall & Aavik, 2016; Gouveia et al., 2015; Milfont et al., 2016; Robinson, 2013; Schuster et al., 2019; Schwartz, 2005; Schwartz et al., 2001; Vecchione et al., 2016). While these trends appear clear at the level of the four higher-order values—albeit with comparatively small effect sizes—they are more heterogeneous and less consistent at the level of the 10 basic values and have not been studied at all at the nuance level.

In addition to investigating these common age trends, which might be largely driven by life stage-specific demands and opportunities (Bardi et al., 2014), as well as biological and psychological aging processes (Schwartz, 2005), some studies examine the impact of personal experiences (e.g., migration; [Bardi et al., 2014; Lönnqvist et al., 2011, 2013], going to college [Bardi et al., 2009], parenthood [Lönnqvist et al., 2018]) and societal and economic events (e.g., the COVID-19 pandemic [Daniel et al., 2022], the 2008 financial crisis [Sortheix et al., 2019], exposure to war [Daniel et al., 2013]) on value development. Taken together, while there is evidence for at least some degree of common and experience-based value development across the lifespan and while scholars have flagged up value development as an emerging research topic that warrants further attention (Schuster et al., 2019), most reviews summarizing the value literature tend to emphasize their stability (rather than change) across the lifespan (Sagiv et al., 2017; Sagiv & Schwartz, 2022; Schuster et al., 2019). Furthermore, the vast majority of value studies are conducted with relatively small and homogeneous samples across relatively short time frames, and all studies reviewed here were conducted at the level of higher-order or basic values (Schuster et al., 2019) as is typical for the field of value research more generally (Sagiv et al., 2017). This leaves value nuances and their dynamics across the lifespan largely unexplored.

Against this background, we argue that value development may be more nuanced than typically represented and propose that a systematic investigation of age-graded differences in personal values from late teenage years to post-retirement across different levels of the value hierarchy (i.e., higher-order values, basic values, value nuances) may reveal underappreciated dynamics.

### The Current Research

In the current research, we examine—both individually and in comparison to other values—the extent to which age differences in personal values can be attributed to different levels of the value hierarchy. To this end, we adopt Schwartz’s circumplex value model (Schwartz, 1992, 1994, 2012), a multi-dimensional value framework that combines 4 higher-order values, 10 basic values, and 20 value nuances—as operationalized through individual items—harnessing a large dataset of over 80,000 individual answers to the Twenty-Item Values Inventory (TwIVI; Sandy et al., 2017). In doing so, we follow previous research (Achaa-Amankwaa et al., 2021; Hang et al., 2021; Mõttus & Rozgonjuk, 2021; Schroeders et al., 2021) and adopt an integrative modeling approach (Hofman et al., 2021) that leverages predictive machine-learning models to advance conceptual understanding (Bleidorn & Hopwood, 2019). This approach is rooted in the notion that predictive approaches may not only maximize prediction but can also enrich exploratory and explanatory approaches by highlighting the practical relevance and real-world meaning of observed patterns and by fostering a deeper understanding of the phenomena in question (Bleidorn et al., 2017; Hofman et al., 2021; Rocca & Yarkoni, 2021).

With this in mind, we predict chronological age from a series of regular- and machine-learning regression models based on (a) 4 higher-order values, (b) 10 basic values, and (c) 20 value nuances. In line with recent personality-development research (Hang et al., 2021; Mõttus & Rozgonjuk, 2021; Schroeders et al., 2021), we do not interpret the resulting prediction models as causal models but rather use them as a statistical tool to locate, quantify, and compare where—within the value hierarchy—age-related information is contained (Stachl et al., 2020).

After discussing the research design and methods, we empirically investigate how values vary across age groups from 18 to 75 years and report the results of three predictive models (i.e., traditional ordinary least squares (OLS) regression, Elastic Net, and M5P Decision Tree) fitted at each level of the value hierarchy (i.e., higher-order values, basic values, and value nuances). We examine the conceptual and methodological relevance of our research findings and close with cautionary remarks. The present study is exploratory and has not been pre-registered. Based on recent conceptually and methodologically similar work on lifespan personality development in intelligence (Schroeders et al., 2021) and the Big Five (Hang et al., 2021; Mottus & Rozgonjuk, 2021), we hypothesize that value nuances will contain more age-sensitive information and hence be more predictive of age than basic human values, which in turn will contain more age-sensitive information and hence be more predictive of age than higher-order values.

## Materials and Methods

### Data-Collection Procedure and Participant Sample

This research uses values and age data from the TIME Magazine Basic Human Values dataset (Du et al., 2023, 2024). The TIME Magazine Basic Human Values Dataset was collected between December 2017 and September 2023. In line with previous projects carried out as part of this research partnership (Ebert et al., 2019; Götz, Bleidorn, & Rentfrow, 2020; Zmigrod et al., 2021), data collection ensued through an interactive online survey, in which participants’ personal values were assessed using scientifically validated measures. The survey (https://time.com/5063406/star-wars-character-quiz/) was launched and promoted via websites and social media channels (e.g., Facebook, Twitter) by TIME Magazine and its media partners (e.g., People, Entertainment Weekly) as a tribute to the global release of the movie “Star Wars: Episode VII—The Last Jedi.” Participants who completed the survey received automatic customized feedback on which Star Wars characters most closely resembled them based on their values. We report all manipulations, measures, and exclusions in these studies (see Online Supplemental Appendix B for a detailed description of the survey). The full analysis code with markdown of results for the current research is available on the OSF (https://osf.io/8sauh/?view_only=5b986a2c970c44ce838ec9c941cd9182). The TIME Sorting Hat Dataset is proprietary and may not be publicly shared but is available upon request from the senior author.

Participants provided informed consent before answering the survey and had the option to receive customized feedback without sharing their data for research purposes. Those who opted in were also asked to answer a short battery of demographic questions (i.e., age, annual income, ethnicity, gender, and place of residence). Overall, completion of the survey took approximately 10 minutes, and after receiving their Star Wars character matches, participants were provided with a more detailed outline of the aims of the associated research project.

The original sample consisted of 122,580 participants. For the current research, we included all participants who self-reported ages between 18 and 75 years and had no missing responses on the value items. The final sample consisted of 80,814 participants, with 57.4% identifying as female, 35.8% identifying as male, and 6.8% reporting other gender identities. The average age was skewed toward younger participants (*M* = 27.5 years; *SD* = 10.32). Age-specific cell sizes ranged from 8,223 participants to 25 participants (see the Data Analysis Strategy section and Online Supplemental Appendices A and C for description and results of the replication with a stratified sample). Of the final sample, 72.7% identified as White, 9.2% as Asian, 8.6% as Hispanic, and 2.4% as Black. The top five countries with the most participants were the United States (*n* = 35,082), the United Kingdom (*n* = 9,061), Canada (*n* = 7,342), Germany (*n* = 3,518), and Australia (*n* = 2,666). As such, the dataset most strongly represents the general U.S. population, of which it is broadly demographically representative (Du et al., 2023).

### Measures

Personal values were assessed using TwIVI (Sandy et al., 2017), a semi-short scale adapting the 40-item Portrait Values Questionnaire (Schwartz, 2003). The TwIVI features 20 portrait-type items (e.g., “Being very successful is important to him or her. S/he likes to impress other people.”), administered on a six-point Likert-type scale on which participants rate how much the described fictional people resemble them (anchors: “not like me at all”; “very much like me”). The TwIVI has been specifically designed for contexts in which semi-short scales are needed, such as the study at hand in which a large-scale sample was recruited through an interactive online survey that would not take up more than 10 minutes. This 20-item scale has been shown to successfully capture the patterns of the longer 40-item Portrait Values Questionnaire, with the average convergence between the TwIVI and standard PVQ measurements being *r* = .91 (Sandy et al., 2017; Vignoles et al., 2018). At the basic value level, Cronbach’s alphas ranged from .29 (*security*) to .79 (*benevolence*), which is (a) comparable to prior findings (Sandy et al., 2017; Schwartz et al., 2001), (b) typical for short scales that emphasize construct breadth and seek to avoid redundancy (Clifton, 2020; Gosling et al., 2003; Rammstedt & John, 2007), and (c) consistent with the notion that value nuances may capture varying amounts of unique information.

### Data Analysis Strategy

We adopted a three-stage analysis approach: Description (Stage 1), Prediction (Stage 2), and Simulation (Stage 3). In the first stage (Description), we charted age-graded differences in personal values across the human lifespan at all three levels of the value hierarchy to provide an exploratory, visual summary of the changes happening across the lifespan on all three levels (i.e., higher-order values, basic values, and value nuances). In the second stage (Prediction), we fitted three models (traditional OLS regression, Elastic Net, and M5P Decision Tree; see Online Supplemental Appendix A for technical details) at each level of the value hierarchy to understand the relationship between value change and age more systematically. In a third and final stage (Simulation), we dove more deeply into scrutinizing the actual predictive abilities of the different hierarchical levels of the personal value system. To do so, we leveraged a simulation-based approach, in which we conducted 10,000 decisions for each of the nine models, identifying the older of two randomly drawn participants based on the personal values they endorse. This last stage translates statistical findings into an intuitive and interpretable metric: the probability that the algorithm correctly identifies the older of the two randomly drawn participants based solely on their personal values. Furthermore, it contextualizes how the accuracy is impacted by the age difference between the participants, as well as the aggregation level of their values.

In the prediction stage, we employed classical econometric, as well as machine-learning models to optimize for interpretability and accuracy. By including linear and non-linear models, we aimed to predict changes accurately, while keeping interpretability in mind. The dataset was split into an 80/20 test and training dataset (Schroeders et al., 2021). Random sampling occurred within each age percentile. The 20% test data therefore had a similar age distribution as the training data but represented a different partition of the data kept separate throughout the training process. This approach—resulting in nine training and nine testing models (each one per model type and value hierarchy layer)—helped prevent both overfitting through cross-validation/out-of-sample testing (Rocca & Yarkoni, 2021; Seeboth & Mõttus, 2018; Yarkoni & Westfall, 2017) and underfitting through comparisons across different models with varying complexity (Jacobucci & Grimm, 2020; Stachl et al., 2020; Yarkoni & Westfall, 2017).

The linear OLS approach was chosen as a baseline for ease of coefficient interpretation. Following previous research (Hang et al., 2021; Mõttus & Rozgonjuk, 2021; Schroeders et al., 2021; Stewart et al., 2022), we predicted age through Elastic Net regressions. Finally, we implemented the M5P algorithm, which produces a decision tree with a linear regression model at each node (Please see Online Supplemental Appendix A for rationales for and introductions to each model choice).

Following Mõttus and Rozgonjuk’s (2021) caution about age predictions being skewed by sample distributions, we replicated all our analyses using a sample stratification approach as a general robustness check. Specifically, we created four age bins, with *n* = 5,900 in each, resulting in a total sample of *N* = 23,600 (age bins: 18–25, 26–33, 34–41, 42–50). We re-ran all analyses and simulations on the age-stratified sample. As the stratification led to substantial data loss, we report findings from the unstratified sample in the main manuscript. However, findings were replicated in the stratified samples, and all analyses with the stratified sample are reported in Tables S1–S3 and Figures S1–S6 in the Online Supplemental Appendix C. Considering the large sample size and novel and exploratory nature of our investigation, we followed conservative guidelines across all analyses for significance testing, with significance thresholds set at *p < .005, **p < .001, ***p < .0001 (Benjamin et al., 2018).

## Results

### Stage 1: Description—Charting Values Across the Lifespan

**Figure 1.**
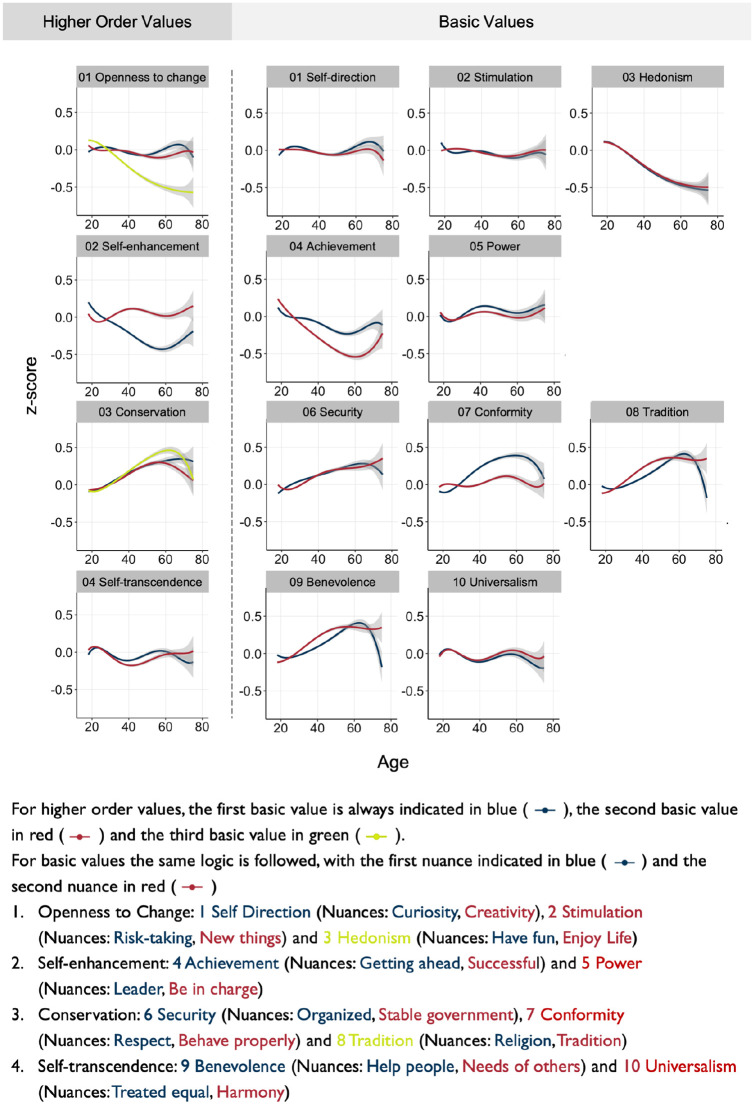
Response for Each Item of the Questionnaire by Age (Polynomial Fit, 95% CI). *Note.* For visualization purposes, we fitted a polynomial line with 95% confidence interval.

Figure 1 shows *z*-standardized scores of value items for each life-year from 18 to 75 grouped by higher-order values, basic values, and value nuances.^2^ At the higher-order level, the observed age trends largely aligned with previous research. That is, *conservation* values increased, and *openness to change* values decreased, and with a smaller magnitude of change, *self-transcendence* values increased, and *self-enhancement* values decreased (Milfont et al., 2016; Robinson, 2013; Schwartz, 2005).

Assessment at the more granular level of basic values suggested both convergence and divergence of basic values that belonged to the same higher-order value. For example, among the *openness to change* values, self-direction and stimulation showed a largely stagnant pattern with greater fluctuations in later years, whereas hedonism showed a continuous decline, likely driving any overall negative associations observed at the higher-order level. Similarly, age-graded differences for the three *conservation* values suggested that, while all tended to increase over the lifespan, increases in *conservation* throughout adulthood may be been driven by different values at different times (i.e., steeper increases in *conformity* and *tradition* precede gains in *security*).

The same approach was used to examine value nuances (i.e., individual items). We saw evidence of both convergence (e.g., *self-direction, stimulation, hedonism*) and divergence (e.g., *achievement, conformity, tradition*) across value nuances that were part of the same basic value. We noted that the increased value variability after the age of 60 needs to be interpreted with caution as it could reflect the reduced data density compared to early adulthood years (Götz, Bleidorn, & Rentfrow, 2020). Taken together, visual inspection of age trends across the three hierarchical value levels indicated pronounced age-graded differences in values across all stages of the human lifespan. It further suggests shared trajectories across the three hierarchical levels of the value system, as well as unique information at each level.

### Stage 2: Predicting Age From Values at Three Hierarchical Levels

**Figure 2.**
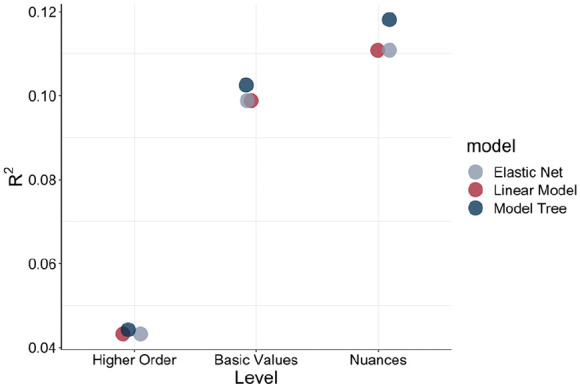
R^2^ Across Model and Level Choices for Test Data. *Note.* R^2^ for nine models—three aggregation levels (higher order, basic value, and nuance level) and three analytical choices (OLS, Elastic Net, and M5P model tree algorithm).

The visual impression that there were shared trajectories across the hierarchical levels of the value systems, as well as unique information at each level, was further supported by our predictive modeling results. Tables 2 (higher-order values), 3 (basic values), and 4 (value nuances) show the training data OLS and Elastic Net coefficients at each of the three levels of the value hierarchy. No traditional *p* values exist for Elastic Net, and no coefficients for the M5P machine-learning algorithm. Figure 2 exhibits R^2^ statistics for the test (i.e., out-of-sample) data.

**Table 2.** Multiple Regression Predicting Age From Higher-Order Values.

Predictors	OLS		Elastic net
	*b*	95% CI	*b*
0. Intercept	27.53***	[27.45, 27.61]	27.53
1. Openness to change	−0.47***	[−0.55, −0.39]	−0.47
2. Self-enhancement	−1.10***	[−1.19, −1.02]	−1.10
3. Conservation	1.74***	[1.66, 1.82]	1.73
4. Self-transcendence	−0.89***	[−0.98, −0.81]	−0.89
Observations	64,653		64,653
R^2^	.042	[0.04, 0.05]	-

*Note.* No traditional *p* values exist for Elastic Net models.

* *p* < .005. **p < .001. ***p < .0001.

**Table 3.** Multiple Regression Predicting Age From Basic Values.

Predictors	OLS		Elastic net
	*b*	95% CI	*b*
0. Intercept	27.52***	[27.45, 27.60]	27.52
1. Self-direction	0.54***	[0.45, 0.62]	0.53
2. Stimulation	0.46***	[0.38, 0.55]	0.46
3. Hedonism	−1.48***	[−1.56, −1.39]	−1.47
4. Achievement	−2.47***	[−2.56, −2.38]	−2.46
5. Power	1.00***	[0.91, 1.08]	0.99
6. Security	1.07***	[0.99, 1.15]	1.07
7. Conformity	0.73***	[0.64, 0.82]	0.72
8. Tradition	0.79***	[0.70, 0.88]	0.79
9. Benevolence	−0.50***	[−0.58, −0.41]	−0.49
10. Universalism	−0.58***	[−0.67, −0.49]	−0.58
Observations	64,653		64,653
R^2^	.096	[.09, .10]	-

*Note.* No traditional *p* values exist for Elastic Net models.

* *p* < .005. **p < .001. ***p < .0001.

**Table 4.** Multiple Regression Predicting Age From Value Nuances.

Predictors	OLS		Elastic net
	*b*	95% CI	*b*
0. Intercept	27.53***	[27.45, 27.60]	27.53
1. Self-direction: Curiosity	0.37***	[0.29, 0.46]	0.37
2. Self-direction: Creativity	0.20***	[0.12, 0.28]	0.20
3. Stimulation: Risk-taking	−0.09	[−0.18, −0.00]	−0.09
4. Stimulation: New things	0.36***	[0.27, 0.45]	0.35
5. Hedonism: Have fun	−0.65***	[−0.74, −0.55]	−0.65
6. Hedonism: Enjoy life	−0.99***	[−1.09, −0.90]	−0.99
7. Achievement: Getting ahead	−0.22***	[−0.32, −0.12]	−0.22
8. Achievement: Successful	−2.42***	[−2.52, −2.33]	−2.42
9. Power: Leader	0.99***	[0.89, 1.09]	−0.98
10. Power: Be in charge	0.03	[−0.07, 0.13]	0.03
11. Security: Organized	0.60***	[0.52, 0.68]	0.60
12. Security: Stable government	0.56***	[0.48, 0.64]	0.56
13. Conformity: Respect	1.12***	[1.03, 1.21]	1.12
14. Conformity: Behave properly	−0.39***	[−0.47, −0.30]	−0.38
15. Tradition: Religion	0.05	[−0.03, 0.13]	0.05
16. Tradition: Tradition	0.93***	[0.84, 1.02]	0.93
17. Benevolence: Help people	−0.62***	[−0.72, −0.52]	−0.61
18. Benevolence: Needs of others	0.15*	[0.05, 0.25]	0.15
19. Universalism: Treating people equal	−0.60***	[−0.69, −0.51]	−0.59
20. Universalism: Harmony	0.10	[0.00, 0.19]	0.09
Observations	64,653		64,653
R^2^	.122	[.12, .13]	−

*Note.* No traditional *p* values exist for Elastic Net models.

* *p* < .005. **p < .001. ***p < .0001.

At the higher-order level, *openness to change* (*b* = −0.47, *p* < .0001), *self-enhancement* (*b* = −1.10, *p* < .0001), and *self-transcendence* (*b* = −0.89, *p* < .0001) were associated with being younger, while *conservation* was associated with being older (*b* = 1.74, *p* < .0001).

At the basic value level, the *openness to change* values *self-direction* (*b* = 0.54, *p* < .0001) and *stimulation* (*b* = 0.46, *p* < .0001) were associated with being older, while hedonism was associated with being younger (*b* = −1.48, *p* < .0001). Among *self-enhancement* values, *power* was associated with being older (*b* = 1.00, *p* < .0001), whereas *achievement* was associated with being younger (*b* = −2.47, *p* < .0001). *Conservation* exhibited strong consistency, with all three composing basic values being associated with being older (*security: b* = 1.07, *p* < .0001; *conformity: b* = 0.73, *p* < .0001; *tradition: b* = 0.79, *p* < .0001), whereas both *self-transcendence* values were associated with being younger (*benevolence: b* = −0.50, *p* < .0001; *universalism; b* = −0.58, *p* < .0001).

At the value nuance level, there were basic values for which nuances were consistently associated with being older or younger. This was the case for both *self-direction* values nuances, which were associated with being older (*curiosity: b* = 0.37, *p* < .0001; *creativity: b* = 0.20, *p* < .0001). Both *hedonism* nuances (*have fun: b* = −0.65, *p* < .0001; *enjoy life: b* = −0.99, *p* < .0001) and *achievement* nuances (*getting ahead: b* = −0.22, *p* < .0001; *successful: b* = −2.42, *p* < .0001) were associated with being younger. Both *security* nuances were associated with being older (*organized: b* = 0.60, *p* < .0001; *stable government: b* = 0.56, *p* < .0001). For *conformity* nuances, the picture was more complex, with *respect* associated with being older (*b* = 1.12, *p* < .0001), and *behave properly* associated with being younger (*b* = −0.39, *p* < .0001). For *benevolence* nuances similarly *needs of others* is associated with being older (*b* = 0.15, *p* = .003), while *helping others* being associated with being younger (*b* = −0.62, *p* < .0001). For each of the remaining basic values, only one of the nuances was significantly associated with age.

**Figure 3.**
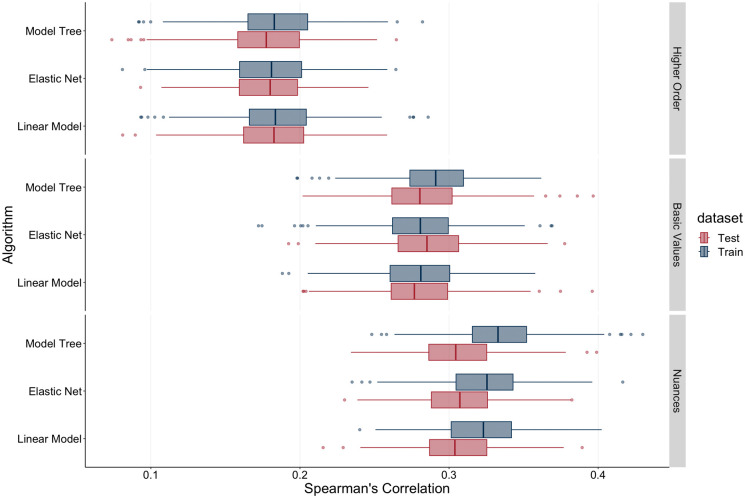
Spearman Correlations Across Model and Level Choices for Test and Training Data. *Note.* Spearman correlations for 500 random samples of 1,000 participants each. Nine different models for training and test data—three aggregation levels (higher order, basic value, and nuance level) across three analytical choices (OLS, Elastic Net, and M5P model tree algorithm).

As a robustness check, we subsequently drew 500 samples, each comprising 1,000 participants (Götz et al., 2021) and calculated the Spearman correlations between actual and predicted age across the nine training and nine test models, resulting in 18 correlation plots (Figure 3). This approach allowed us to assess whether the observed relationship between actual and predicted age would replicate across smaller, randomly drawn subsets of data. By averaging these correlations and constructing confidence intervals, we ensured that the reported effects were not driven by idiosyncrasies in the full sample but represented a stable and replicable pattern. Consistent with the results described earlier, we found that the Spearman correlations between actual age and predicted age rose with increasing value granularity, with the out-of-sample correlations being *r_s_* = .18 (*p* < .0001; OLS regression), *r_s_* = .18 (*p* < .0001; Elastic Net), and *r_s_* = .18 (*p* < .0001; M5P model tree algorithm) at the higher-order value level; *r_s_* = .28 (*p* < .0001; OLS regression), *r_s_* = .28 (*p* < .0001; Elastic Net), and *r_s_* = .28 (*p* < .0001; M5P model tree algorithm) at the basic value level; and *r_s_* = .31 (*p* < .0001; OLS regression), *r_s_* = .31 (*p* < .0001; Elastic Net), and *r_s_* = .31 (*p* < .0001; M5P model tree algorithm) at the value nuance level. Moreover, we observed that while the performance (measured through correlations between predicted and actual age) was slightly higher for the M5P model tree algorithm in the training dataset than for the other approaches, in the test dataset, performance was very slightly lower at the higher-order level. Performance was, however, very similar across the OLS, Elastic Net, and M5P model tree algorithm approaches. Overall, we submit that the improvement in performance may be primarily attributed to analysis granularity, rather than the usage of cutting-edge analysis methods.

### Stage 3: Contextualizing Findings and Benchmarking Predictive Accuracy

**Figure 4.**
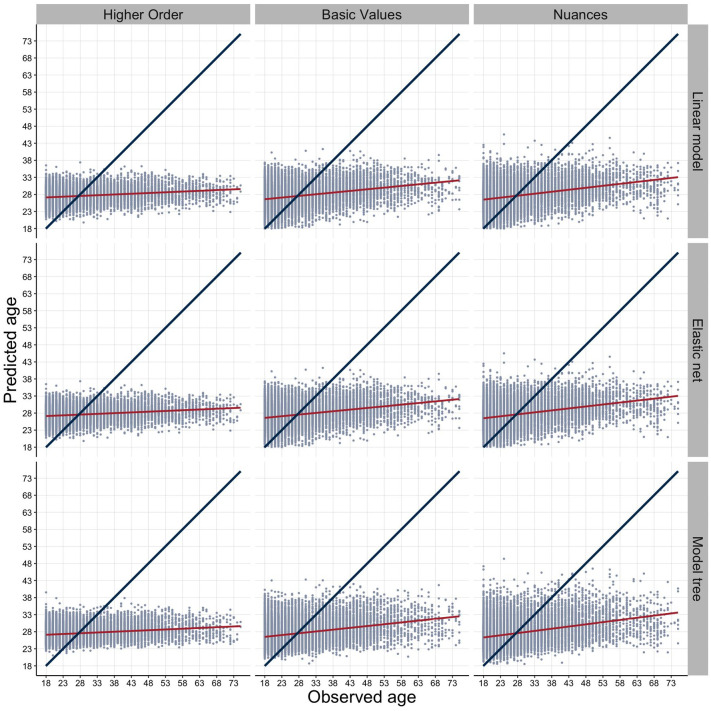
Observed Versus Predicted Age Across Model and Level Choices for Test Data. *Note.* Observed versus predicted age for testing data. Nine different models—three aggregation levels (higher order, basic value, and nuance level) and three analytical choices (OLS, Elastic Net, and M5P model tree algorithm). The blue line indicates the y = x curve, which benchmarks perfect fit (i.e., 100% accuracy in age prediction). The red line displays linear fit through the predictions (which were partly derived from non-linear models).

In the third stage of our analysis, we aimed to contextualize our findings and cast the age-sensitive information from each level of the value hierarchy into practical, accessible terms. We plotted the test dataset predictions for each of the nine models against actual participant age (Figure 4).

**Figure 5.**
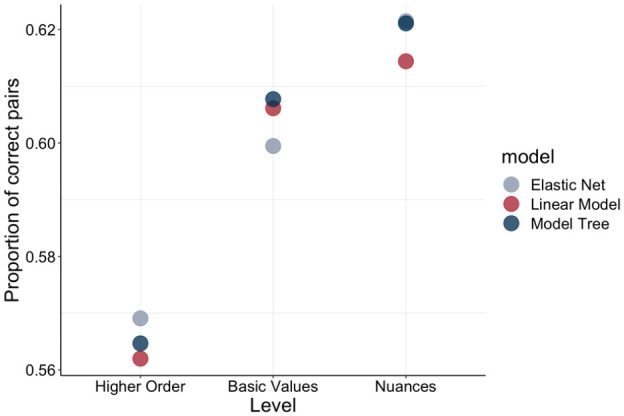
Proportion of Correct Predictions Across Model and Level Choices for Test Data. *Note.* Proportion of correct paths across nine different models—three aggregation levels (higher order, basic value, and nuance level) and three analytical choices (OLS, Elastic Net, and M5P model tree algorithm).

Given the striking discrepancies between predicted versus actual age, we conducted a simple simulation to further understand the observed patterns. We randomly drew two participants and let the algorithms predict each individual’s age, dichotomously encoding whether the algorithm successfully predicted which participant was older (Achaa-Amankwaa et al., 2021). If two drawn participants had the same real age, they were discarded from the analyses. We drew 10,000 pairs for each level (i.e., higher-order values, basic values, and value nuances) and each analysis method (OLS, Elastic Net, and M5P decision tree approach), resulting in 90,000 decisions. Figure 5 depicts accuracies across hierarchical value levels and model choices.

In keeping with the aforementioned results, accuracy increased with value granularity. Across models, higher-order value predictions performed ~6% above chance level (which is 50% for binary decisions), basic value prediction accuracies performed ~10% above chance level, and value nuances performed ~12% above chance level.

**Figure 6.**
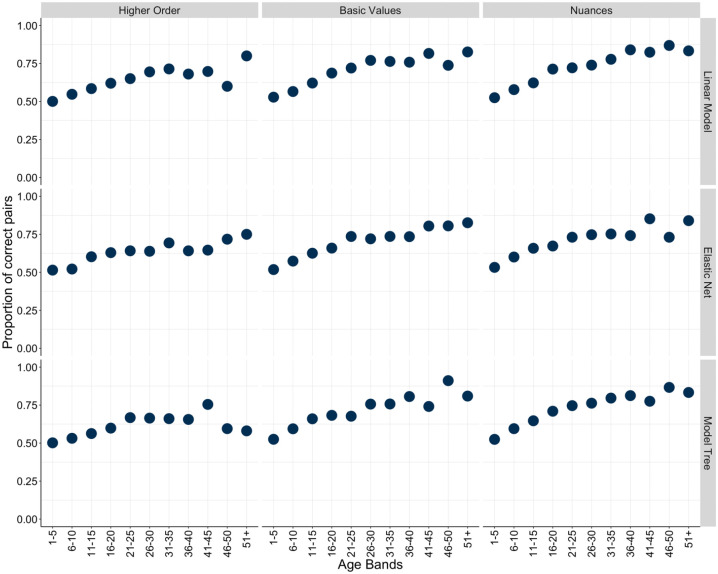
Proportion of Correct Predictions by Age Across Model and Level Choices for Test Data. *Note.* Age-difference-graded proportion of correct decisions across nine different models—three aggregation levels (higher order, basic value, and nuance level) and three analytical choices (OLS, Elastic Net, and M5P model tree algorithm).

To better understand the abilities and limits of the different algorithms to predict age based on value information, we charted the prediction success rate as a function of the age difference of the two randomly drawn individuals (Figure 6). We noted that algorithms fared better when predicting participant seniority among pairs with larger age differences. That is, while basic values and—to a greater extent—value nuances continued to consistently outperform predictions based on higher-order values across model choices and hierarchical value levels, accuracies at or above 70% were only achieved for randomly drawn pairs with age differences larger than 20 years. Practically speaking, this means that while the values measured here cannot be used to predict the exact age of individuals, they can be used to effectively infer who is likely to be older based on the values they hold.

## General Discussion

We drew from a large-scale online sample and adopted an integrative modeling approach (Hofman et al., 2021) to examine (a) whether age-graded differences in personal values emerge across the lifespan and, if so, (b) at which level of the value hierarchy these differences are most pronounced. To that end, in Stage 1 (Description), we plotted age-graded differences in higher-order values, basic values, and value nuances from age 18 to 75 years. Then, in Stage 2 (Prediction), we employed three different modeling approaches (i.e., OLS regressions, Elastic Net regularization, M5P decision trees) to predict individuals’ age based on personal values at each of the three hierarchical values. Finally, in Stage 3 (Simulation), we conducted a series of additional analyses to further explore the practical meaning of our findings and better contextualize the observed effects.

**Figure 7.**
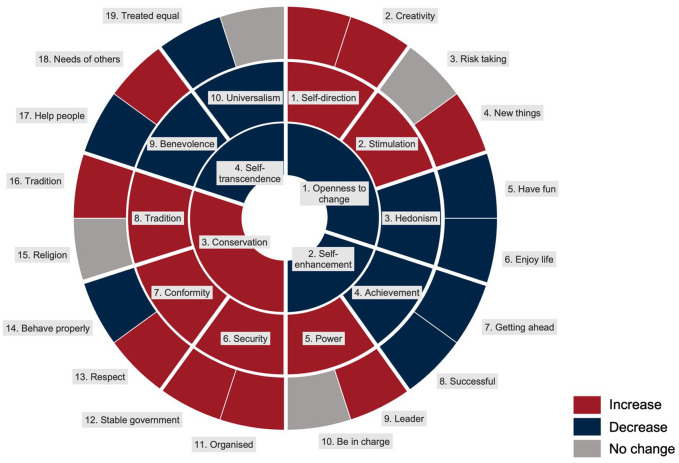
Summary of Age-Graded Differences Throughout the Lifespan in Schwartz Human Values Across Three Hierarchical Levels. *Note.* Visual summary of change across the lifespan, red indicating positive coefficients, blue negative coefficients, and gray not significant coefficients. The outer circle shows the value nuance coefficients, the middle circle shows basic values, and the inner circle shows the higher-order values.

Across all analytical steps and algorithmic models, we found consistent support for our hypothesis (see Figure 7). That is—mirroring prior research on personality development (Big Five; Hang et al., 2021; Mõttus & Rozgonjuk, 2021, intelligence; Schroeders et al., 2021)—values exhibit systematic age-graded differences across all stages of the lifespan. We further showed that value nuances contained more age-sensitive information and had greater predictive power than basic values, which in turn contained more age-sensitive information and greater predictive power than higher-order values.

Specifically, in the first, descriptive stage, our initial plotting of lifespan age-graded differences across higher-order values, basic values, and value nuances suggests both shared trajectories and unique patterns at each of the hierarchical levels of the value system. That is, the age-graded differences observed among the higher-order values (i.e., increasing *conservation* and—to a lesser extent—*self-transcendence* as well as decreasing *openness to change* and—to a lesser extent—*self-enhancement*) dovetail well with previous value-development research across diverse global populations (Milfont et al., 2016; Robinson, 2013; Schuster et al., 2019; Schwartz, 2005) and broader personality-development patterns, such as the maturity principle (Bleidorn et al., 2013; Roberts et al., 2008). At the same time, shifting to basic values and—even further—to value nuances reveals both alignment and discrepancies within value groups. This finding not only highlights the added utility of considering more granular value systems but may also help to explain and reconcile some of the existing inconsistencies within the broader value-development literature (Schuster et al., 2019).

In the second, predictive stage, the formal quantification of the relationships between values and age indicated that *conservation* was most strongly and positively related to age, whereas the other three higher-order values were negatively related to age (see Figure 7). This trend is particularly surprising for *self-transcendence* because, in general, we would have expected older participants to put greater emphasis on social values (e.g., *benevolence* and *universalism*; Carstensen & Reynolds, 2023; Fung & Carstensen, 2003). At the basic value level, *self-direction* (*openness to change*), *stimulation* (*openness to change*), *power* (*self-enhancement*), *security* (*conservation*), *conformity* (*conservation*), and *tradition* (*conservation*) were positively associated with age, whereas *hedonism* (*openness to change*), *benevolence* (*self-transcendence*), *universalism* (*self-transcendence*), and *achievement* (*self-enhancement*) were negatively associated with age. A similar picture emerged at the value nuance level, where *curiosity* (*self-direction*), *creativity* (*self-direction*), *new things* (*stimulation*), *leader* (*power*), *organized* (*security*), *stable government* (*security*), *respect* (*conformity*), and *tradition* (*tradition*) were positively associated with age, while *have fun* (*hedonism*), *enjoy life* (*hedonism*), *getting ahead* (*achievement*), *being successful* (*achievement*), *behave properly* (*conformity*), *help people* (*benevolence*), and *treated equal* (*universalism*) were negatively associated with age. *Risk taking* (*stimulation*), *being in charge* (*power*), *religion* (*tradition*), *needs of others* (*benevolence*), and *harmony* (*universalism*) were not statistically associated with age. Taken together, these findings suggest both convergence and divergence within basic and higher-order value groupings, thus underscoring the unique developmental information that resides at each of the three levels of the value hierarchy. Of note, the incremental predictive power was substantial, with value nuances accounting for about three times as much variance in age as higher-order values (4.4% versus 12.1%).

From a conceptual perspective, some of our observed broad and specific age-graded differences in personal values provide empirical support for prior theorizing on adaptive aging (Heckhausen et al., 2010). For example, a strong decline in valuing *achievement* as individuals mature may indicate that once individuals have families and have established themselves professionally (as many individuals do at that stage of their life), they become less concerned with their own accomplishments (Schwartz et al., 2001; Veroff et al., 1984). Relatedly, the consistent positive associations between value nuances pertaining to *conformity, security*, and *tradition* and age may reflect people’s increasing commitment to habitual patterns and existing social networks as they grow older (Glenn, 1974; Schwartz et al., 2001; Tyler & Schuller, 1991). In other words, as people become older, they become more concerned with inhabiting stable and predictable environments (Schwartz, 2005) and the preservation—rather than growth—of personal resources or status (Robinson, 2013).

In our third and final analysis step, we sought to further contextualize the observed effects (Funder & Ozer, 2019; Götz et al., 2022) and translate them into accessible, practical terms by highlighting the actual meaning of our predictive power (Rocca & Yarkoni, 2021; Yarkoni & Westfall, 2017). We found that exact age predictions were not accurate across models and hierarchical value levels, but our models were fairly accurate in differentiating younger from older participants based on their values. In the random pairwise participant comparisons, models could predict the older participant with accuracies ranging from just above chance level 55.47% (higher order–Elastic Net) to 61.47% (value nuances–Elastic Net). Accuracies generally improved as more granular value nuances were considered and as the age gap between participants increased. The value nuance performance averaged 80.93% for the four highest age-gap bands of 36–40, 41–45, 46–50, and 51+.

### Research Contributions and Implications

Our paper offers conceptual and methodological contributions to the values- and personality-development literature at large.

First—in conjunction with mounting evidence from other constructs (Hang et al., 2021; Mõttus & Rozgonjuk, 2021; Schroeders et al., 2021)—our findings suggest that age-graded variation in personality does occur along many dimensions at different hierarchical levels and that to get the complete picture of personality development, we will need to consider not only the broad domains that we are most familiar with but also the small nuances that they consist of. To be clear, like others who have reported similar findings for other components of personality, we are neither denying the relevance, legitimacy, and parsimony of higher-order constructs nor arguing for a radical shift wherein we will always and only consider personality nuances. Rather, we think of this as a bandwidth-fidelity tradeoff (John et al., 1991; Mõttus & Rozgonjuk, 2021; Rentfrow, 2010) and simply caution researchers to deliberately choose the level of the personality hierarchy that best corresponds to their research questions and goals, rather than defaulting to the broadest hierarchical levels. Whenever possible, we recommend not choosing at all, but rather reporting multiple hierarchical levels in parallel, so as to maximize the information that can be gained. We note that this argument—and the incremental power of personality nuances—is not limited to personality development. Similar empirical findings and theoretical arguments are emerging across personality science, from life outcomes (Seeboth & Mõttus, 2018; Stewart et al., 2022) and geographical ambiance (Elleman et al., 2020) to culture (Achaa-Amankwaa et al., 2021).

On a methodological level, we note that while we did our best to produce accurate estimates, bringing together large samples, cross-validation, regularization, and cross-model comparisons (Del Giudice, 2021; Mõttus et al., 2020; Stachl et al., 2020; Yarkoni & Westfall, 2017), and while we yielded what is conventionally regarded as sizable correlations between predicted ages and participants’ reported ages (Funder & Ozer, 2019; Gignac & Szodorai, 2016), the actual ability of our models to predict participants’ actual age was relatively weak. This may surprise some readers—and certainly surprised us at first—but is actually not uncommon, even with stronger correlations than ours. As Stachl and colleagues (2020, p. 618) put it: “An almost perfect correlation can be found even when predictions are off in absolute terms by a large degree.” This also becomes apparent when engaging in the sobering exercise of trying to use correlations to draw meaningful conclusions about specific individuals (Mõttus, 2022)—as attempted in our pairwise comparisons. Our point here is not to say that this means the current research is uninformative. On the contrary, we believe that if anything, our integrative modeling approaches enriched the descriptive goals of our work. However, we think this is a potent reminder to heed the advice of other scholars to seize the opportunities that predictive modeling affords to benchmark our findings and scrutinize their real-world applicability (Bleidorn et al., 2017; Bleidorn & Hopwood, 2019; Rocca & Yarkoni, 2021; Yarkoni & Westfall, 2017). If used in this way, integrative modeling may enable us to better understand the reach and limits of our theories and findings (Hofman et al., 2021) and may helpfully contribute to the ongoing discussion in the field of how to determine the meaning and relevance of empirical effects (Anvari et al., 2023; Anvari & Lakens, 2021; Funder & Ozer, 2019; Götz et al., 2022, 2024). Indeed, the actual age correlations we find across the higher-order, basic, and nuance value levels are all on par with—or even larger than—those reported in the Big Five literature when using measures of similar length, such as the Big Five Inventory 2 (BFI-2) (Hang et al., 2021).

Furthermore, while we observed a considerable increase in accuracy when moving toward more fine-grained value nuances, we note that across analytical applications, a simple OLS-based linear regression model achieved statistical out-of-sample performances that were comparable—and at times even slightly superior—to the considerably more sophisticated and complex machine-learning models (i.e., Elastic Net, M5P Decision Tree). While this may be surprising, it is a regular occurrence (Christodoulou et al., 2019; Del Giudice, 2021; Jacobucci & Grimm, 2020) that should caution researchers against the blind use of advanced machine-learning models and highlights the utility of cross-model comparisons.

### Limitations and Future Research Directions

Our study has several limitations. First, as the study of personality nuances is only just emerging (Hang et al., 2021; Mõttus & Rozgonjuk, 2021; Schroeders et al., 2021), the TwIVI scale we used was originally designed to measure higher-order and basic values, not value nuances (Sandy et al., 2017). There is currently no formally developed value nuance framework. This means that the items used may not be the most suitable or representative set of value nuances. It may also mean that our findings underestimate the predictive power that could be reached with a more comprehensive, carefully selected set of value nuances (Stewart et al., 2022). Future research should develop a systematic, hierarchical value taxonomy (Condon et al., 2020) that encompasses a more deliberately selected and wider set of nuances.

Second, our research was based on a self-selected online survey. While it is large, diverse, and regionally representative within the United States (Du et al., 2023), it skews toward younger participants (mean age = 27.4 years; *SD* = 10.26). This sample resembles similar samples obtained in large-scale online data-collection efforts such as the Gosling-Potter Personality internet Project (Gosling et al., 2004); mean age in unrestricted original sample = 27.6 (Gosling et al., 2004), the TIME Magazine Sorting Hat Dataset, (Ebert et al., 2019; Götz, Bleidorn, & Rentfrow, 2020; Götz, Stieger, et al., 2020; mean age = 27.3, *SD* = 10.3), the Synthetic Aperture Personality Assessment (SAPA; Condon et al., 2017; Elleman et al., 2020; median age = 25), and Project Implicit (Xu et al., 2014; mean age = 27.23). Furthermore, as with most research in social-personality psychology, our research is predominantly based on U.S. samples, which limits findings’ generalizability (Thalmayer et al., 2021). While we leverage a comparatively diverse sample, including non-U.S. participants from the United Kingdom (*n* = 9,061), Canada (*n* = 7,342), Germany (*n* = 3,518), and Australia (*n* = 2,666), our research is no exception to this general trend. As such, we acknowledge that the findings reported here should be interpreted with caution, when applied in context outside of the United States. We hope that future research will consolidate and extend our work, thus expanding its global reach.

Third, our study was restricted to cross-sectional data, which means that we inferred rather than directly observed value change and that we are unable to rule out the possibility of confounding cohort effects (Schaie, 1977), for example, individuals who were in their 60s in 2020, were born in the 1960s, and came of age in the 1980s at the height of capitalism, during the “greed is good” era. This upbringing might have instilled in them an achievement-focused mind-set that prizes self-interest above all else. Although longitudinal designs come with their own set of limitations, such as attrition effects, time-of-measurement effects, and self-selection effects (Robinson, 2013; Schaie, 1996), and although the personality-development literature has so far observed a clear convergence between findings from cross-sectional and longitudinal studies (Roberts & Yoon, 2022), future research should aim to provide further longitudinal evidence for the stability and change of values at all hierarchical levels across the lifespan. Such work may then fruitfully inform—and be informed by—evolving theoretical models of value change, such as the dual route model of value change (Bardi & Goodwin, 2011), as well as feed into theoretical models of how values affect behavior, such as Sagiv and Roccas’ (2021) process model.

Finally, aside from its specific content focus, on a methodological level, the current research illustrates the power of big data and machine learning to make inferences about individuals and their personal characteristics. Of note, the present work itself may be a comparatively innocuous demonstration of that—requiring individuals to proactively select into (a) providing informed consent, (b) completing a 20-item self-report personal values questionnaire, and (c) opting in to donate their data in order for us to study what their personal values reveal about their age. However, other work has shown that far more easily obtainable data—such as digital footprints on social media, including Facebook likes, natural language on Twitter, and headshots—can be used to accurately infer highly intimate and sensitive personal attributes, such as personality, political ideology, or sexual orientation (Kosinski et al., 2013, 2024; Park et al., 2015; Youyou et al., 2015). In this new world of big data, machine learning, and—increasingly developing—generative artificial intelligence, ethical sensitivity is thus of paramount importance (Alexander et al., 2020; Kosinski et al., 2015), and while it is crucial not to fall prey to the false dichotomy of “privacy versus insight” (Matz et al., 2022), it is just as crucial for researchers to ascertain that the collection, storage, analysis, and interpretation of their data are in line with state-of-the-art ethical, legal, and professional standards. Then—and likely only then—can research live up to its mandate of beneficence, generating novel insights that benefit citizens, while protecting their anonymity and privacy in the process.

## Conclusions

The current research brought together large-scale data and an integrative modeling approach to examine how age-related information is distributed across three hierarchical levels of personal values. Our results suggest that value nuances capture considerable unique age-sensitive information, above and beyond basic values and higher-order values, which also manifests in improved predictive performance. As such, the present work contributes to the field in three ways; that is, by extending the literature on personality development, personal values, and personality nuances. In conjunction with a fast-growing body of knowledge that highlights their utility and relevance (Achaa-Amankwaa et al., 2021; Condon et al., 2020; Elleman et al., 2020; Hang et al., 2021; Mõttus et al., 2017, 2019; Mõttus & Rozgonjuk, 2021; Revelle et al., 2020; Seeboth & Mõttus, 2018; Stewart et al., 2022; Wessels et al., 2020), the current findings underscore that nuances are here to stay as formal and full members of the values hierarchy that matters not only for values and development but also for personality science as a whole.

## Supplemental Material

sj-docx-1-psp-10.1177_01461672241312570 – Supplemental material for Human Values Across the Lifespan: Age-Graded Differences at Three Hierarchical Levels and What We Can Learn From Them Supplemental material, sj-docx-1-psp-10.1177_01461672241312570 for Human Values Across the Lifespan: Age-Graded Differences at Three Hierarchical Levels and What We Can Learn From Them by Andrés Gvirtz, Matteo Montecchi, Amy Selby and Friedrich M. Götz in Personality and Social Psychology Bulletin

## Notes

1. For ease of communication, throughout this manuscript, we will refer to “shifts,” “increases,” and “decreases” but would like to ask readers to bear in mind that we are not reporting on personality development in a strict within-person sense, but rather on age-graded differences based on between-person comparisons.

2. We would like to note that a long-standing discussion in the value research literature concerns the appropriate use of standardization when dealing with value scores. Because values are always positively keyed (i.e., there are no reverse values) and because values operate within a system in which opposing or conflicting values guide trade-offs (e.g., self-enhancement versus self-transcendence), some scholars have proposed that researchers should use ipsatization (i.e., centering values within each person) to control for scale use (e.g., see the study by Schwartz, 2003). While there is merit in this approach, we have decided against the use of ipsatization. This decision has been informed by various thoughtful critiques, arguing that the potential costs of using ipsatization might not be outweighed by its benefits (Fischer, 2004; Hicks, 1970) as pointedly summarized by Fischer & Milfont (2010): “. . . standardization may address some of the problems caused by different response styles. However, we also noted that it is hard to establish whether scores are being affected by response styles. For research purposes, it is better to model such response style factors and estimate the effect directly [. . .] rather than controlling for it without knowing the exact extent and nature of it.” Instead, we standardize at the level of analyses, meaning for every facet, basic, and higher-order value, we create *z*-scores, thus enabling straightforward comparison of age-graded differences.
