# Supplementary material for: Human Values Across the Lifespan: Age-Graded Differences at Three Hierarchical Levels and What We Can Learn From Them
Source: Pers Soc Psychol Bull. 2025 Feb 5;52(6):1482–501. doi: 10.1177/01461672241312570 (PMC13096630; doi:10.1177/01461672241312570)
Supplement: sj-docx-1-psp-10.1177_01461672241312570 – Supplemental material for Human Values Across the Lifespan: Age-Graded Differences at Three Hierarchical Levels and What We Can Learn From Them [file sj-docx-1-psp-10.1177_01461672241312570.docx]

**Web Appendix**

**Human Values Across the Lifespan:**

**Age-Graded Differences at Three Hierarchical Levels and What We Can Learn from Them**

| **Table of Contents** |
| --- |
| Web Appendix A: Methodological detail appendix – Description of model choices 2 |
| Web Appendix B: Methodological detail appendix – Description of survey 3-5 |
| Web Appendix C: Robustness check: Stratified replication of all analyses and simulations 6-14 |
| Web Appendix D: Works cited in the web appendix 16-17 |

**Web Appendix A: Methodological Detail Appendix (MDA) – Description of model choices**

We adopted three modelling approaches (traditional OLS regression, Elastic Net, and M5P Decision Tree) and implemented them in parallel. We chose classical econometric and machine-learning models to optimize for interpretability and accuracy. The linear OLS approach was chosen as a baseline for ease of coefficient interpretation. Following guidelines from previous research (Hang et al., 2021; Mõttus & Rozgonjuk, 2021; Schroeders et al., 2021; Stewart et al., 2022), we predicted age through Elastic Net regressions, a method that combines the regularization penalties from LASSO (least absolute shrinkage and selection operator; (Tibshirani, 1996) and ridge regressions, alleviating weaknesses associated with each of them alone (Chapman et al., 2016). LASSO penalizes the absolute value of coefficients, setting the irrelevant values to 0. Ridge regressions (Hoerl & Kennard, 1970) penalize the magnitude of the regression coefficients, shrinking correlated predictors towards each other (Friedman et al., 2010) and less important predictors close to but not at 0 (i.e., reducing their impact rather than eliminating them). Elastic net regressions combine both methods, producing a more parsimonious model while working well with highly inter-correlated predictors (Zou & Hastie, 2005). Regulations were chosen using 10-fold cross-validation (Hang et al., 2021; Mõttus & Rozgonjuk, 2021; Stewart et al., 2022; Yarkoni & Westfall, 2017). Lastly, we implemented the M5P algorithm, which produces a decision tree with a linear regression model at each node. While to the best of our knowledge, the M5P algorithm has not been previously used in psychology research, it has shown promising results in environmental and urban science (Shaban et al., 2016; Zhan et al., 2011). Specifically, the M5P algorithm creates subgroups and models them individually through linear regressions, allowing potentially non-linear trends between values and age to be modeled through a collection of linear approximations (Hornik et al., 2009; Quinlan, 1992; Wang & Witten, 1996; Witten et al., 2011)

**Web Appendix B: Methodological Detail Appendix (MDA) – Description of survey**

The following are brief descriptions, as well as the actual survey questions of the TIME Magazine Basic Human Values Dataset. There are four psychological constructs measured, specifically (1) human values (2) self-esteem, (3) agentic narcissism and (4) subjective misfit. This research focused on the basic human values. In addition, five sociodemographic constructs were assessed, (1) age, (2) gender, (3) location, (4) ethnicity and (5) income.

**Psychological Constructs**

1. **Basic human values (Sandy, Gosling, Schwartz & Koelkebeck, 2017).**

The inventory encompasses 20 items, whereby participants are instructed to rate how much they resemble the characterization of a fictional person, using a 6-point Likert-scale, ranging from 6 “very much like me” to 1 “not like me at all”. At the basic value level, Cronbach’s alphas ranged from .29 (*security*) to .79 (*benevolence*), which is i) comparable to prior findings (Sandy et al., 2017; S. H. Schwartz et al., 2001), ii) typical for short scales that emphasize construct breadth and seek to avoid redundancy (Clifton, 2020; Gosling et al., 2003; Rammstedt & John, 2007), and iii) consistent with the notion that value nuances may capture varying amounts of unique information. The items read as follows:

*(1) This person believes they should always show respect to their parents and to older people. It is important to them to be obedient*

*(2) Religious belief is important to them. They try hard to do what their religion requires.*

*(3) It's very important to this person to help the people around them. This person wants to care for their well-being.*

*(4) This person thinks it is important that every person in the world be treated equally. They believe everyone should have equal opportunities in life.*

*(5) This person thinks it's important to be interested in things. They like to be curious and to try to understand all sorts of things.*

*(6) This person likes to take risks. They are always looking for adventures.*

*(7) This person seeks every chance they can to have fun. It is important to them to do things that give them pleasure.*

*(8) Getting ahead in life is important to this person. This person strives to do better than others.*

*(9) This person always wants to be the one who makes the decisions. They like to be the leader.*

*(10) It is important to them that things be organized and clean. They really do not like things to be a mess.*

*(11) It is important to this person to always behave properly. This person wants to avoid doing anything people would say is wrong.*

*(12) This person thinks it is best to do things in traditional ways. It is important to this person to keep up the customs they have learned.*

*(13) It is important to this person to respond to the needs of others. This person tries to support those they know.*

*(14) This person believes all the worlds' people should live in harmony. Promoting peace among all groups in the world is important to them.*

*(15) Thinking up new ideas and being creative is important to this person. This person likes to do things in their own original way.*

*(16) This person thinks it is important to do lots of different things in life. They always look for new things to try.*

*(17) This person really wants to enjoy life. Having a good time is very important to them.*

*(18) Being very successful is important to this person. This person likes to impress other people.*

*(19) It is important to them to be in charge and tell others what to do. They want people to do what they say.*

*(20) Having a stable government is important to this person. This person is concerned that the social order be protected.*

**Socio-Demographics**

We assessed (1) age, (2) gender, (3) location, measured through ZIP code of residence (within US) and state of residence (within US), (4) ethnicity and (5) annual income (in USD 50k steps).

**Web Appendix C: Robustness Check: Stratified replication of all analyses and simulations**

Following Mõttus & Rozgonjuk (2021), who cautioned against the risk of prediction bias towards younger age due to skewed sample distributions, we repeated all our analyses using a sample stratification approach as a general robustness check. Specifically, we created four age bins, with n = 5,900 in each, resulting in a total sample of N = 23,600 (age bins: 18 - 25, 26 - 33, 34 - 41, 42 - 50). We re-ran all analyses and simulations on the age-stratified sample. As the stratification leads to substantial data loss (i.e., 70.8%; from N = 80,814 to N = 23,600) we reported findings from the unstratified sample in the main manuscript, and report findings from the stratified sample here, in the web appendix, in Table S1 to Table S3 and Figure S1 to Figure S6. The findings from the main manuscript were replicated successfully across the robustness checks.

**Figure S1**

*Response for Each Item of the Questionnaire by Age (Polynomial Fit, 95% CI)***
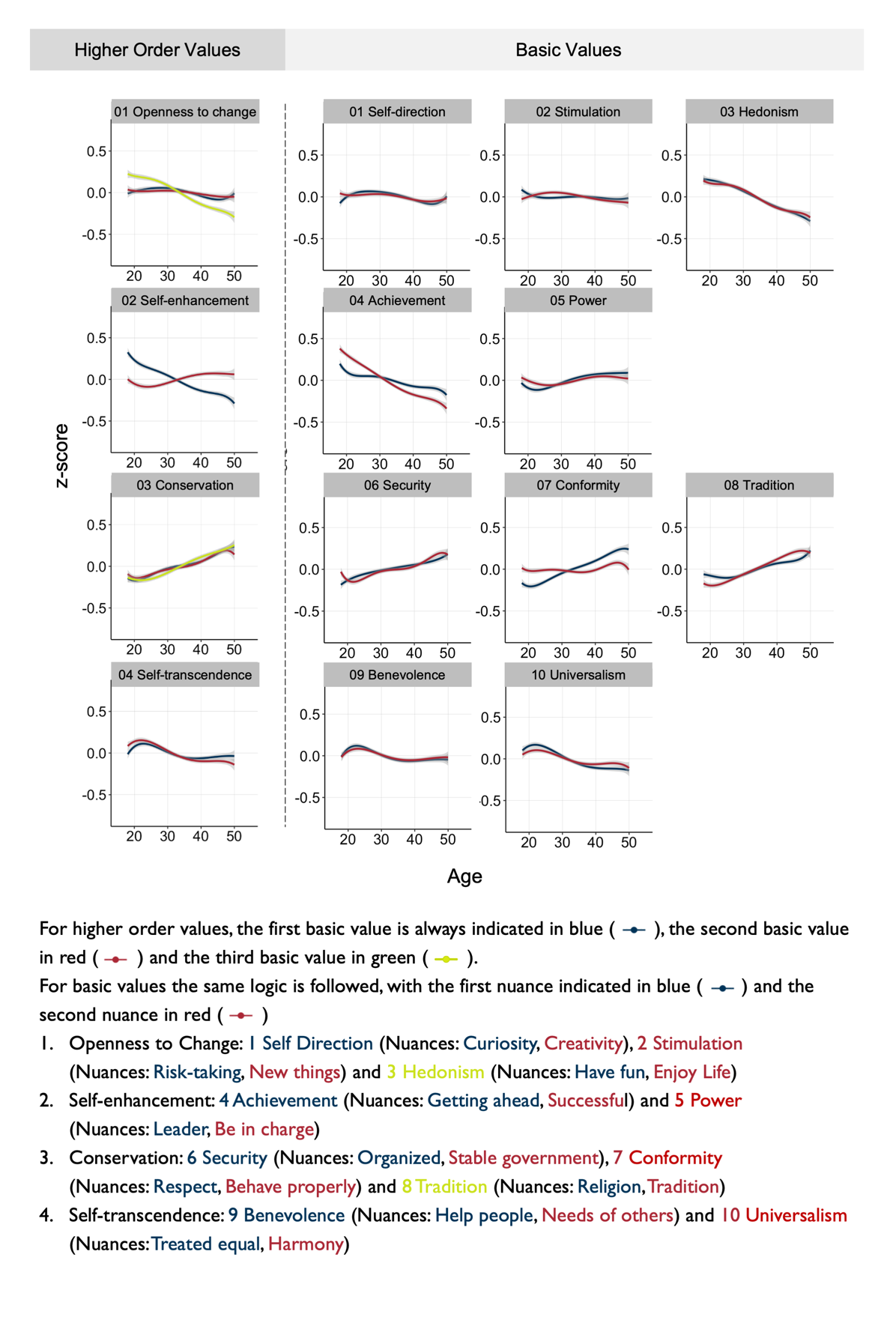
**

**Figure S2**

*Stratified Sample: R^2^ across Model and Level Choices for Test Data*

*
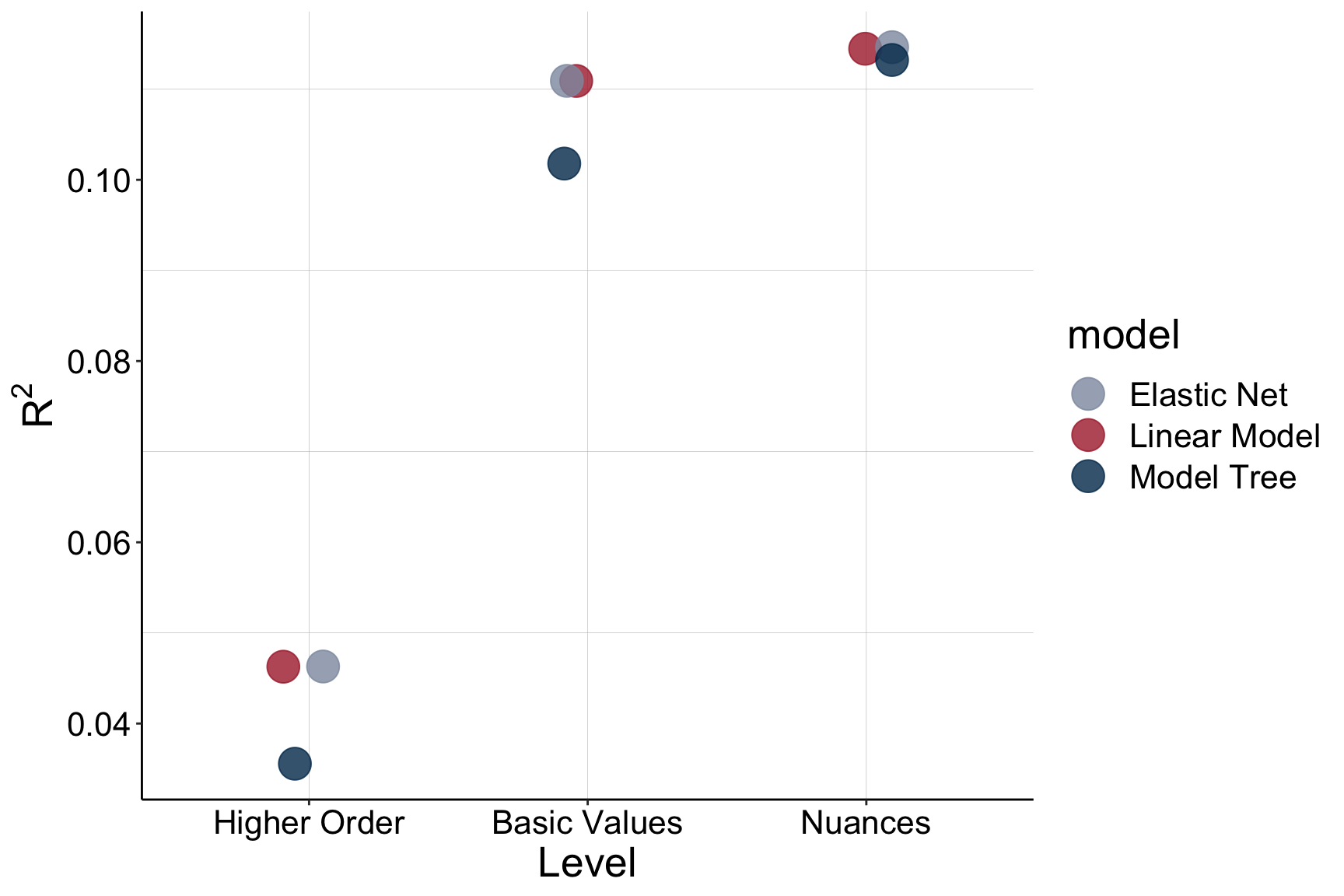
*

*Note.* R^2^ for nine models–three aggregation levels (higher-order, basic value and nuance level) and three analytical choices (OLS, Elastic Net and M5P model tree algorithm).

**Figure S3**

Stratified Sample: Spearman Correlations across Model and Level Choices for Test and Training Data

**
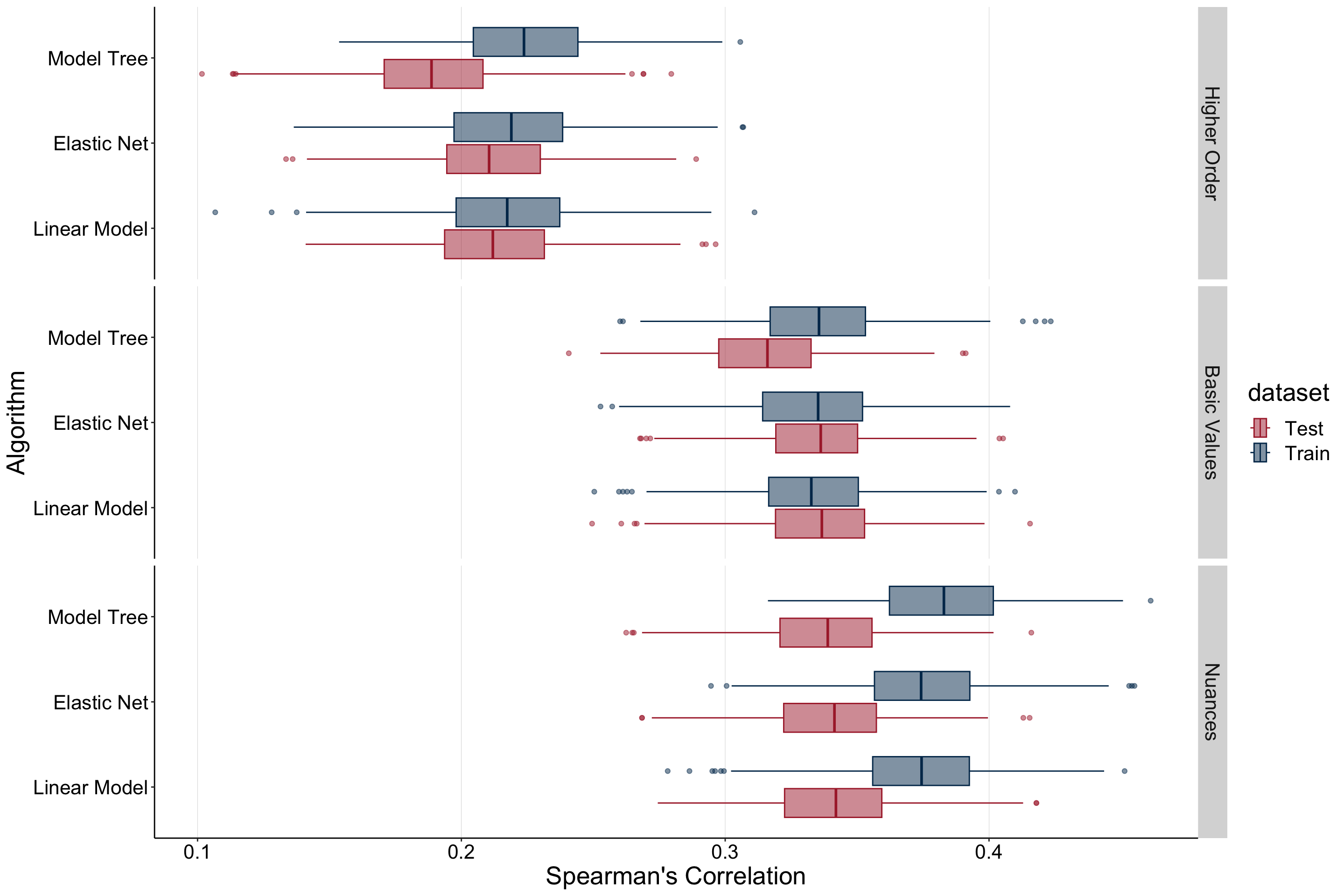
**

*Note.* Spearman correlations for 500 random samples of 1,000 participants each. Nine different models for training and test data–three aggregation levels (higher-order, basic value and nuance level) across three analytical choices (OLS, Elastic Net and M5P model tree algorithm).

**Figure S4**

*Stratified Sample: Observed versus Predicted Age across Model and Level Choices for Test Data*

**
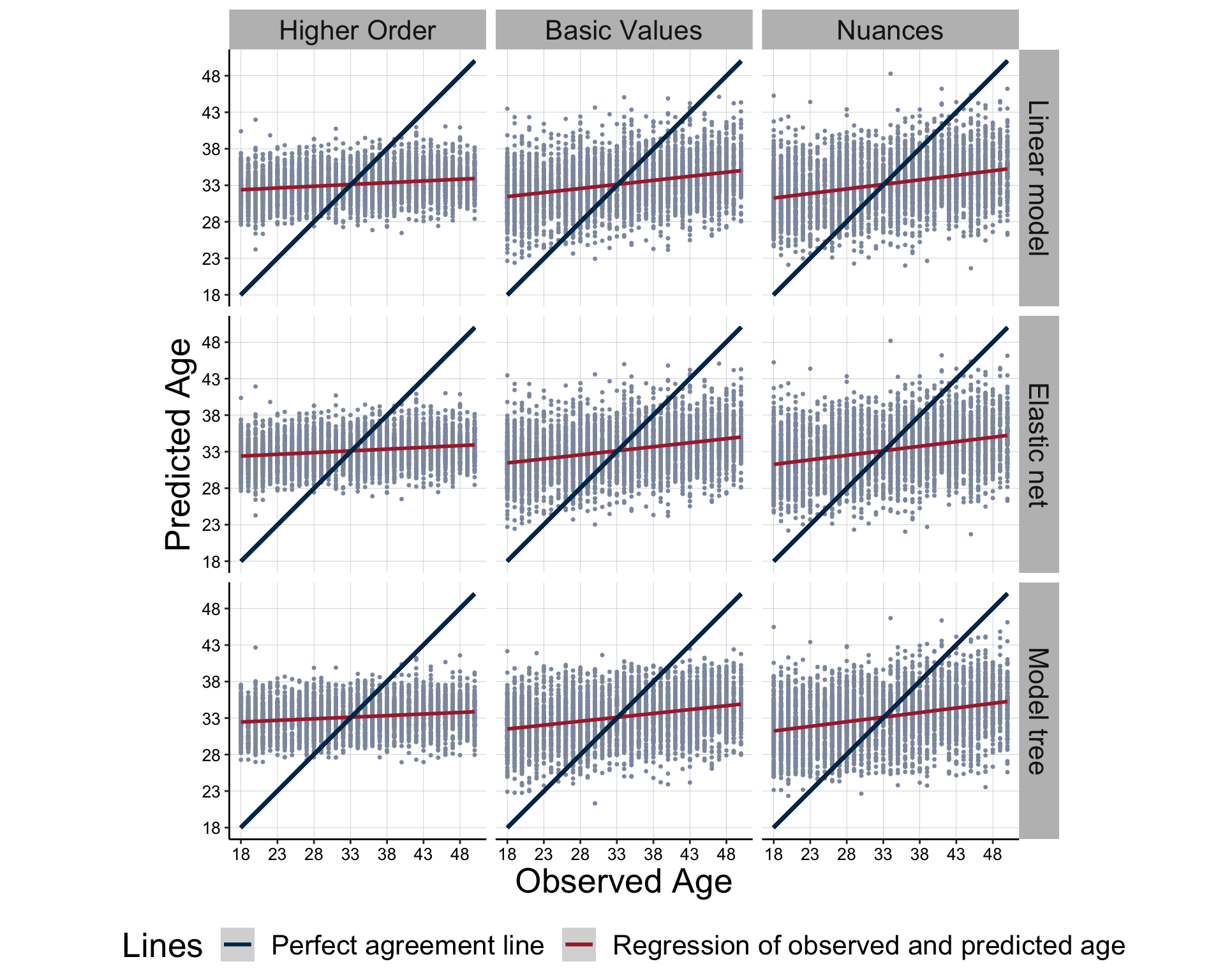
**

*Note.* Observed versus predicted age for testing data. Nine different models—three aggregation levels (higher order, basic value, and nuance level) and three analytical choices (OLS, Elastic Net, and M5P model tree algorithm). The blue line indicates the y = x curve, which benchmarks perfect fit (i.e., 100% accuracy in age prediction). The red line displays linear fit through the predictions (which were partly derived from non-linear models).

**Figure S5**

*Stratified Sample: Proportion of Correct Predictions across Model and Level Choices for Test Data*

**
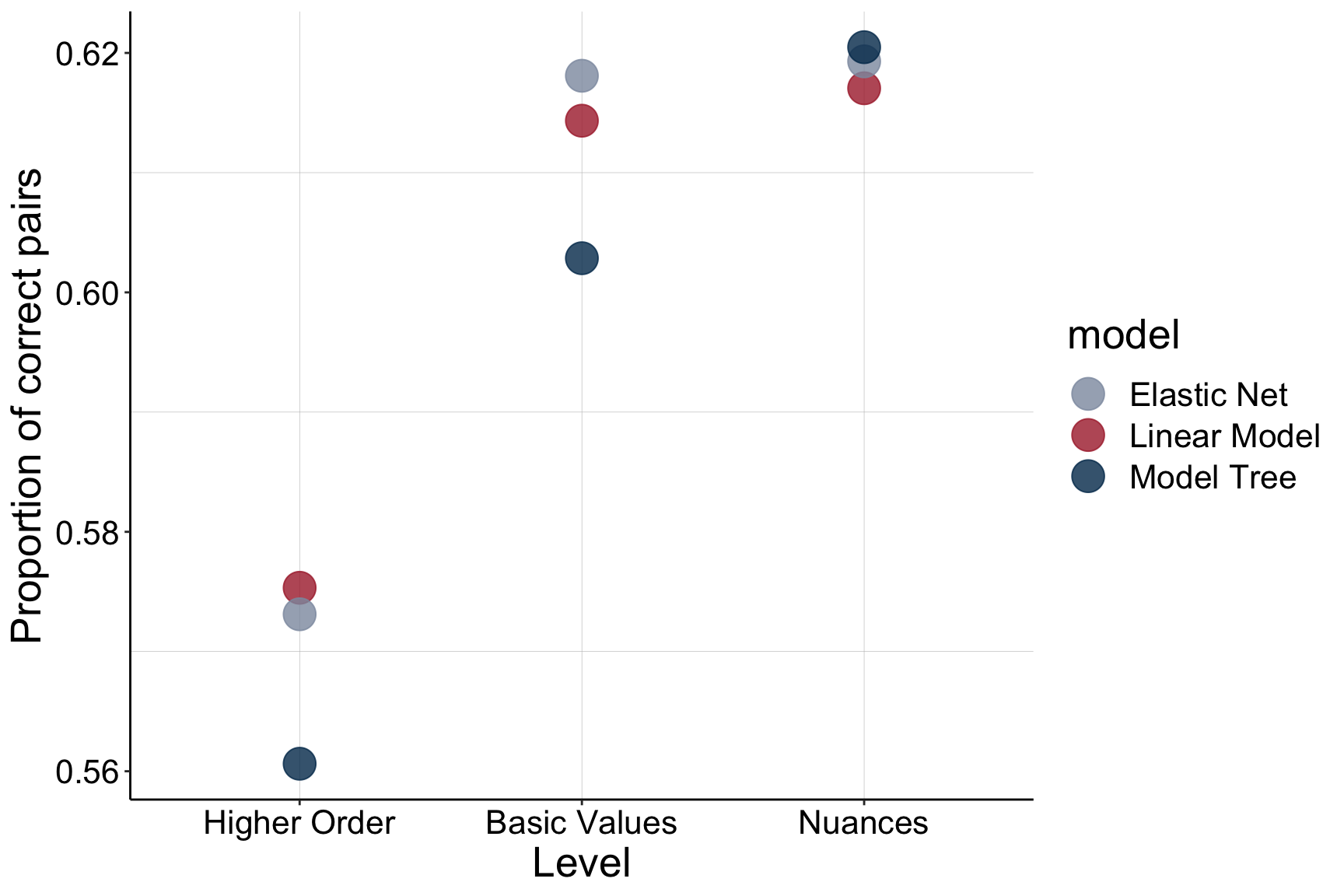
**

*Note.* Proportion of correct paths across nine different models—three aggregation levels (higher order, basic value, and nuance level) and three analytical choices (OLS, Elastic Net, and M5P model tree algorithm).

**Figure S6**

*Stratified Sample: Proportion of Correct Predictions by Age across Model and Level Choices for Test Data*


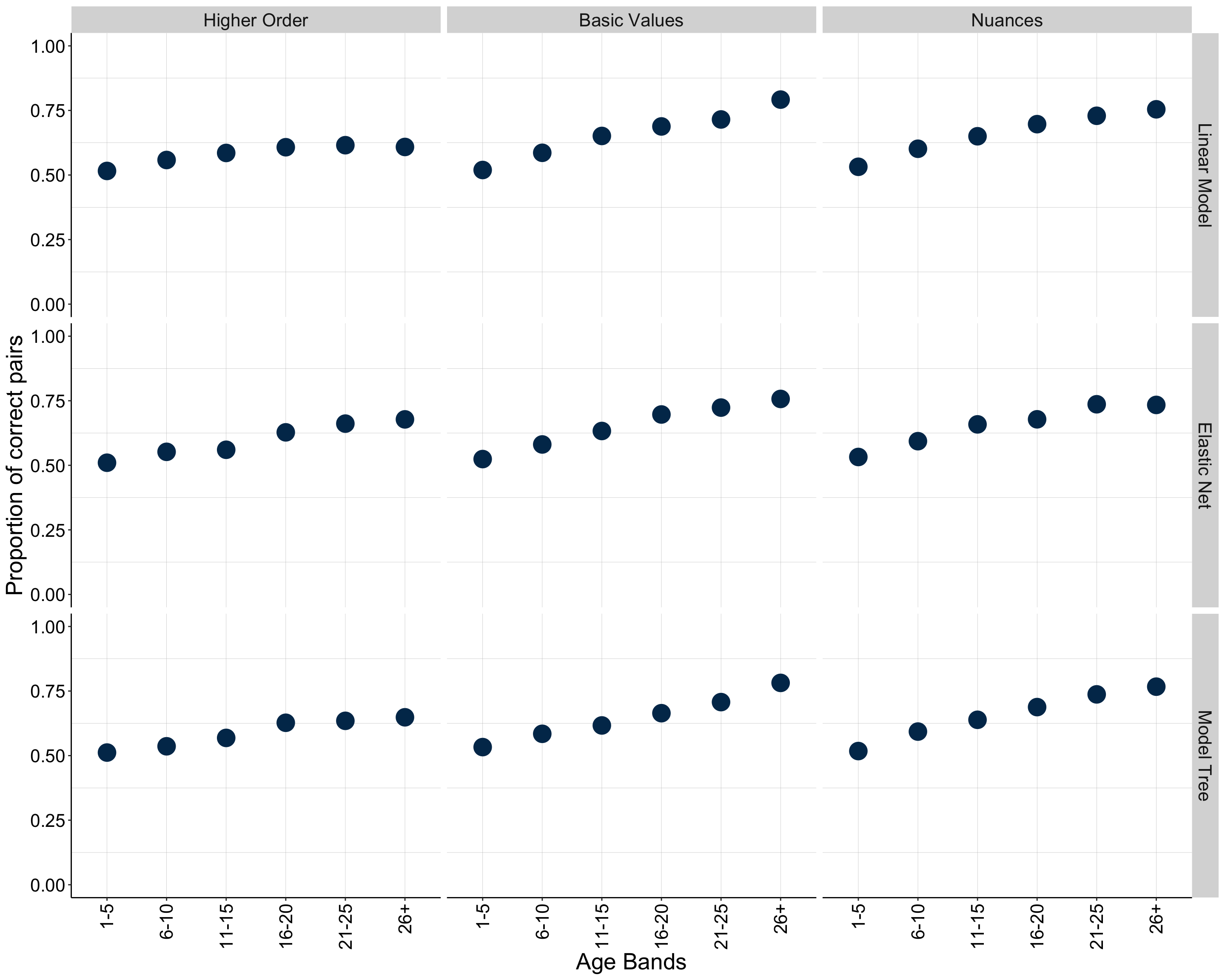


*Note.* Age differentiated proportion of correct paths across nine different models– three aggregation levels (higher-order, basic value and nuance level) times three analytical choices (OLS, Elastic Net and M5P model tree algorithm).

**Table S1**

*Stratified SampleL Multiple Regression Predicting Age from Higher-Order Values*

| Predictors | OLS | | Elastic Net | |
| --- | --- | --- | --- | --- |
|  | *b* | 95% CI | *b* | |
| 0. Intercept | 33.12*** | [32.99, 33.25] | 33.12 | |
| 1. Openness to change | -0.36*** | [-0.50, -0.22] | -0.36 | |
| 2. Self-enhancement | -1.07*** | [-1.21, -0.92] | -1.06 | |
| 3. Conservation | 1.70*** | [1.56, 1.84] | 1.69 | |
| 4. Self-transcendence | -1.13*** | [-1.27, -0.99] | -1.12 | |
| Observations | 18,880 |  | 18,800 | |
| R^2^ | .049 | [.04,.06] | - | |
| *Note.* No traditional *p* values exist for Elastic Net models  **p* < .005. ***p* < .001. ****p* < .0001 | | | |  |

**Table S2**

*Stratified SampleL Multiple Regression Predicting Age from Basic Values*

| Predictors | OLS | | Elastic Net | |
| --- | --- | --- | --- | --- |
|  | *b* | 95% CI | *b* | |
| 0. Intercept | 33.13*** | [33.00, 33.25] | 33.13 | |
| 1. Self-direction | 0.26** | [0.12, 0.41] | 0.26 | |
| 2. Stimulation | 0.59*** | [0.44, 0.74] | 0.58 | |
| 3. Hedonism | -1.22*** | [-1.36, -1.08] | -1.22 | |
| 4. Achievement | -2.51*** | [-2.67, -2.36] | -2.50 | |
| 5. Power | 1.18*** | [1.03, 1.33] | 1.17 | |
| 6. Security | 1.06*** | [0.92, 1.20] | 1.05 | |
| 7. Conformity | 0.82*** | [0.67, 0.98] | 0.81 | |
| 8. Tradition | 0.66*** | [0.51, 0.81] | 0.66 | |
| 9. Benevolence | -0.45*** | [-0.60, -0.31] | -0.45 | |
| 10. Universalism | -0.87*** | [-1.02, -0.72] | -0.87 | |
| Observations | 18,800 |  | 18,800 | |
| R^2^ | .109 | [.10,.12] | - | |
| *Note.* No traditional *p* values exist for Elastic Net models  **p* < .005. ***p* < .001. ****p* < .0001 | | | |  |

**Table S3**

*Stratified SampleL Multiple Regression Predicting Age from Value Nuances*

| Predictors | OLS | | | Elastic Net |
| --- | --- | --- | --- | --- |
|  | *b* | 95% CI | *b* | |
| 0. Intercept | 33.13*** | [33.00, 33.25] | 33.13 | |
| 1. Self-direction: Curiosity | 0.12 | [-0.03, 0.26] | 0.11 | |
| 2. Self-direction: Creativity | 0.17 | [0.03, 0.31] | 0.17 | |
| 3. Stimulation: Risk-taking | 0.12 | [-0.04, 0.27] | 0.12 | |
| 4. Stimulation: New things | 0.41*** | [0.25, 0.56] | 0.40 | |
| 5. Hedonism: Have fun | -0.42*** | [-0.58, -0.25] | -0.42 | |
| 6. Hedonism: Enjoy life | -0.94*** | [-1.11, -0.77] | -0.94 | |
| 7. Achievement: Getting ahead | -0.45*** | [-0.61, -0.28] | -0.44 | |
| 8. Achievement: Successful | -2.35*** | [-2.51, -2.19] | -2.35 | |
| 9. Power: Leader | 1.15*** | [0.98, 1.32] | 1.15 | |
| 10. Power: Be in charge | 0.11 | [-0.20, 0.16] | 0.10 | |
| 11. Security: Organized | 0.54*** | [0.41, 0.67] | 0.53 | |
| 12. Security: Stable government | 0.71*** | [0.57, 0.84] | 0.70 | |
| 13. Conformity: Respect | 1.09*** | [0.94, 1.24] | 1.08 | |
| 14. Conformity: Behave properly | -0.24** | [-0.39, -0.10] | -0.24 | |
| 15. Tradition: Religion | 0.01 | [-0.13, 0.15] | 0.01 | |
| 16. Tradition: Tradition | 0.83*** | [0.68, 0.98] | 0.83 | |
| 17. Benevolence: Help people | -0.52*** | [-0.69, -0.35] | -0.52 | |
| 18. Benevolence: Needs of others | -0.03 | [-0.20, 0.13] | -0.03 | |
| 19. Universalism: Treated equal | -0.71*** | [-0.87, -0.55] | -0.71 | |
| 20. Universalism: Harmony | -0.12 | [-0.28, 0.04] | -0.12 | |
| Observations | 18,800 |  | 18,800 | |
| R^2^ | .138 | [.13,.15] | - | |
| *Note.* No traditional *p* values exist for Elastic Net models  **p* < .005. ***p* < .001. ****p* < .0001 | | | | |

**Web Appendix D: Works cited in the web appendix**

Chapman, B. P., Weiss, A., & Duberstein, P. R. (2016). Statistical learning theory for high dimensional prediction: Application to criterion-keyed scale development. *Psychological Methods*, *21*(4), 603–620. https://doi.org/10/f9pvpc

Friedman, J., Hastie, T., & Tibshirani, R. (2010). Regularization paths for generalized linear models via coordinate descent. *Journal of Statistical Software*, *33*(1). https://doi.org/10/bb3d

Hang, Y., Soto, C., Speyer, L. G., Haring, L., Lee, B., Ostendorf, F., & Mõttus, R. (2021). Age differences in the personality hierarchy: A multi-sample replication study across the life span. *Journal of Research in Personality*, *93*, 104121. https://doi.org/10/gkfx26

Hoerl, A. E., & Kennard, R. W. (1970). Ridge regression: Biased estimation for nonorthogonal problems. *Technometrics*, *12*(1), 55–67. https://doi.org/10/cznwqw

Hornik, K., Buchta, C., & Zeileis, A. (2009). Open-source machine learning: R meets weka. *Computational Statistics*, *24*(2), 225–232. https://doi.org/10/frm9km

Mõttus, R., & Rozgonjuk, D. (2021). Development is in the details: Age differences in the big five domains, facets, and nuances. *Journal of Personality and Social Psychology*, *120*(4), 1035–1048. https://doi.org/10/ghkt4s

Quinlan, J. R. (1992). *Learning with continuous classes*. https://www.semanticscholar.org/paper/Learning-With-Continuous-Classes-Quinlan/ead572634c6f7253bf187a3e9a7dc87ae2e34258

Schroeders, U., Watrin, L., & Wilhelm, O. (2021). Age-related nuances in knowledge assessment. *Intelligence*, *85*, 101526. https://doi.org/10/gqgsvg

Shaban, K. B., Kadri, A., & Rezk, E. (2016). Urban air pollution monitoring system with forecasting models. *IEEE Sensors Journal*, *16*(8), 2598–2606. https://doi.org/10/f8fhnm

Stewart, R. D., Mõttus, R., Seeboth, A., Soto, C. J., & Johnson, W. (2022). The finer details? The predictability of life outcomes from big five domains, facets, and nuances. *Journal of Personality*, *90*(2), 167–182. https://doi.org/10/gqgsvw

Tibshirani, R. (1996). Regression shrinkage and selection via the lasso. *Journal of the Royal Statistical Society: Series B (Methodological)*, *58*(1), 267–288. https://doi.org/10/gfn45m

Wang, Y., & Witten, I. H. (1996). *Induction of model trees for predicting continuous classes* [Working Paper]. https://researchcommons.waikato.ac.nz/handle/10289/1183

Witten, I. H., Frank, E., & Hall, M. A. (2011). *Data mining: Practical machine learning tools and techniques* (3rd ed.). Morgan Kaufmann Publishers Inc.

Yarkoni, T., & Westfall, J. (2017). Choosing prediction over explanation in psychology: Lessons from machine learning. *Perspectives on Psychological Science*, *12*(6), 1100–1122. https://doi.org/10/gcmrmp

Zhan, C., Gan, A., & Hadi, M. (2011). Prediction of lane clearance time of freeway incidents using the M5P tree algorithm. *IEEE Transactions on Intelligent Transportation Systems*, *12*(4), 1549–1557. https://doi.org/10/cjqnsz

Zou, H., & Hastie, T. (2005). Regularization and variable selection via the elastic net. *Journal of the Royal Statistical Society: Series B (Statistical Methodology)*, *67*(2), 301–320. https://doi.org/10/b8cwwr
